# Hidden palette: culturing the sauropsid gut microbiome reveals a high prevalence and diversity of bacteria that undergo carotenoid biosynthesis

**DOI:** 10.1093/femsec/fiag100

**Published:** 2026-09-07

**Authors:** Alix E Matthews, Victoria Gates, Kartikey Sharma, Sebastian Gallego, Madeline Desmond, Samantha S Fontaine, Brian K Trevelline, Marcella D Baiz

**Affiliations:** Department of Biological Sciences, University at Buffalo, State University of New York, Buffalo, NY 14260, United States; Department of Biological Sciences, University at Buffalo, State University of New York, Buffalo, NY 14260, United States; Department of Biological Sciences, University at Buffalo, State University of New York, Buffalo, NY 14260, United States; Department of Biological Sciences, University at Buffalo, State University of New York, Buffalo, NY 14260, United States; Department of Biological Sciences, Kent State University, Kent, OH 44242, United States; Department of Biological Sciences, Kent State University, Kent, OH 44242, United States; Department of Biology, University of South Dakota, Vermillion, SD 57069, United States; Department of Biological Sciences, Kent State University, Kent, OH 44242, United States; Department of Biological Sciences, University at Buffalo, State University of New York, Buffalo, NY 14260, United States

**Keywords:** birds, carotenoids, host-microbe interactions, lizards, microbial culturing, pigmentation

## Abstract

Pigments play essential roles in communication, physiology, and ecological adaptation in taxa across the tree of life and are especially important for animals, where carotenoid pigments contribute to diverse functions including coloration, antioxidant defense, and immune function. One potentially important yet largely overlooked source of carotenoids is host-associated microbes. Microbially derived carotenoids may influence host phenotype and physiology, but the extent to which they occur or vary across hosts remains largely unknown. To begin to address this gap, we isolated gut-associated bacteria from multiple wild sauropsids. We cultured 157 distinct bacterial isolates from 25 individuals representing eight host species; 21% of isolates were yellow- or orange-pigmented and 40% exhibited absorbance spectra consistent with the presence of carotenoids. 16S rRNA gene sequencing revealed that isolates spanned four phyla and 35 genera. Isolates that were consistent with the presence of carotenoids were found across all four phyla, with *Pantoea* being the most common carotenoid-associated genus. Host species did not significantly explain variation in isolate community composition. These results demonstrate that carotenoid-capable bacteria are both taxonomically diverse and widespread among gut-associated bacteria from wild sauropsids. More broadly, our findings provide insight into a hidden dimension of host-microbe interactions and their potential function.

## Teaser

Gut bacteria that produce carotenoids are common and diverse in wild birds and lizards, suggesting that they may be a hidden source of pigments with potential functional and ecological consequences.

## Introduction

Natural pigments underlie functionally important phenotypes in taxa across the tree of life. For example, chlorophyll, a green pigment found in plants, cyanobacteria, and algae, is essential for initiating photosynthesis. The colorful plumage of many bird species is also produced by pigments such as carotenoids, melanins, and porphyrins (among others); these pigment-based plumages are critical for sexual signaling and other types of communication (Hill 1991, Hedley and Caro 2022). Some micro-organisms can also express colorful pigment-based phenotypes which are functionally important and can sometimes be pronounced; for instance, the pinkish color of hypersaline lakes is attributed to carotenoid pigments synthesized by halophilic microbes in the water, where these molecules play key roles in photoprotection and antioxidant defense (Teller 1987, Mandelli et al. 2012). However, unless grown in culture, the color phenotypes of most microbes are unknown. This is especially true for host-associated communities, where microbes are dispersed within complex ecological communities and remain hidden from view. Although many microbes in such diverse communities may still express pigments, their presence is often overlooked. Furthermore, culture-independent techniques commonly used for host-associated microbial studies (e.g. DNA metabarcoding) do not indicate functional activity, even if presence is detected. Thus, the proximate and ultimate functions of pigmented microbes remain poorly understood. In particular, the ecological, evolutionary, and functional roles of microbial pigments in host-microbe interactions are largely unexplored, despite their potential to mediate important host phenotypic traits, such as those involved in communication and sexual signaling.

Unlike bacteria, most animals cannot synthesize carotenoid pigments *de novo* and must obtain them from external sources (Toews et al. 2017). Carotenoid acquisition has therefore traditionally been assumed to occur from diet alone (Hill 1992), including diets supplemented with microbially produced carotenoids in commercial feeds (Pasarin and Rovinaru 2018, Al-Monofy et al. 2025). Recent research highlights a potential link between host-associated carotenoid-synthesizing microbes and host phenotypes. For example, correlations between the presence of carotenoid-producing bacterial taxa and variation in host coloration have been reported in both invertebrates, including malacostracans (Koellsch et al. 2024) and bivalves (Liu et al. 2020), as well as vertebrates like fish (Nguyen et al. 2020) and birds (Martínez-Renau et al. 2024). In birds specifically, the abundance of certain bacterial taxa in the gut microbiome predicted to encode carotenoid biosynthesis genes (e.g. *Sphingomonas*) were found to be positively associated with the extent of carotenoid-based yellow plumage color of their hosts, suggesting a potential microbial contribution to host coloration (Baiz et al. 2024). Beyond their role in coloration, carotenoids also provide several important physiological functions to hosts. Carotenoids can act as antioxidants by mitigating oxidative stress (Stahl and Sies 2003), contribute to vitamin A metabolism as precursors of retinoids (Grune et al. 2010), and influence host immune function (Blount et al. 2003). Due to trade-offs in the allocation of carotenoids to signal phenotypes versus physiological maintenance, carotenoid-based coloration is widely thought to be an honest indicator of mate quality (Hill 1991, Freeman-Gallant et al. 2011). Whether (and to what extent) bacterial carotenoids contribute to host coloration, immunity, or reproductive success remains unknown, leaving a key gap in our understanding of the microbiome’s role in host fitness and sexual selection. Addressing this gap first requires characterizing the diversity and estimating the prevalence of host-associated microbes that are capable of synthesizing carotenoids.

Bacterial taxa across at least 12 phyla are known to synthesize carotenoids, producing more than 300 distinct compounds (Yabuzaki 2017). Some well-characterized carotenoid producers include human pathogens such as *Staphylococcus aureus*, which produces staphyloxanthin (Pelz et al. 2005) and draws its specific epithet from its golden color (Rosenbach 1884). They fill various ecological roles and are found in diverse environments, from marine ecosystems (Du et al. 2006) to extreme Antarctic habitats (Dieser et al. 2010), as well as host-associated microbiomes (Branden et al. 1980, Yoon et al. 2011, Kim et al. 2015). Several bacterial taxa commonly found in host-associated microbiomes are predicted to synthesize carotenoids, including *Pseudomonas, Rhizobium*, and *Sphingomonas* (Escallón et al. 2019, Baiz et al. 2024). Given the broad taxonomic and ecological distribution of carotenoid-producing bacteria and the complexity of their biosynthetic pathways, carotenoids may be widely advantageous for bacteria to synthesize. Indeed, carotenoids prevent photodamage and oxidative stress (López et al. 2023), protect cells from extreme environmental stressors (Dieser et al. 2010), and even act as virulence factors (Liu et al. 2005). However, the roles and possible advantages of carotenoid biosynthesis by symbiotic bacteria in the host gut microbiome remain uncharacterized. Evaluating the prevalence and diversity of carotenoids synthesized by gut symbionts is a critical first step to understand how microbial carotenoids may influence host fitness and the stability of host-microbe symbioses.

Here, we isolated phenotypically unique bacteria from the gut microbiomes of eight sauropsid host species to study their pigment phenotypes and genotypic diversity. We mainly focused our sampling on colorful wood warblers (family Parulidae), though we also included two avian species which do not express carotenoid-pigmented plumage as well as one lizard species with variation in pigmentation to broaden the evolutionary representation of hosts. Our primary objectives were to (1) characterize the phenotypes and genotypes of gut bacteria across host species, and (2) test the hypothesis that the host gut microbiome includes bacteria capable of carotenoid biosynthesis. We examined bacterial diversity by reconstructing the phylogenetic history of our isolates and further tested whether they exhibit host- or environment-specific distributions.

## Methods

### Fieldwork

We collected fecal samples from 20 wild birds between 03–27 June 2024 across five sites in western New York State, USA. Avian hosts included five species within the family Parulidae: Blue-winged Warbler (BWWA; *Vermivora cyanoptera*), Common Yellowthroat (COYE; *Geothlypis trichas*), Chestnut-sided Warbler (CSWA; *Setophaga pensylvanica*), Hooded Warbler (HOWA; *Setophaga citrina*), and Yellow Warbler (YEWA; *Setophaga petechia*), one species within the family Mimidae: Gray Catbird (GRCA; *Dumetella carolinensis*); and one species within the family Hirundinidae: Purple Martin (PUMA; *Progne subis*). We captured individuals in a mist net (parulids and Gray Catbirds) or at the nest box (Purple Martins) and immediately placed them in a sterile sample collection chamber made of a paper bag lined with aluminum foil which sat underneath a ¼ inch steel mesh platform separating the bird from the fallen fecal material. After 10 min, we removed the bird from the chamber and collected whole feces by using sterile inoculating loop to transfer it into a tube containing 750 µl of 30% glycerol in PBS. After returning to the laboratory at the University at Buffalo, we vortexed the sample tubes and transferred half of the volume to a second tube and froze both tubes at −80°C until they were used for bacterial cultivation. We also collected a field negative control by sampling an empty sterile collection chamber in the same manner as described above.

We captured adult wild common wall lizards (POMU; *Podarcis muralis*) on 06 June 2024 from urban, roadside stone walls in Cincinnati, Ohio, USA using a thread lasso attached to an extendable fishing rod. We placed each lizard into individual nylon, breathable cloth sacks and then placed all sacks into a cooler for transport to the laboratory at Kent State University. In the laboratory, we housed lizards in individual plastic rat cages (18″ L × 9.25″ W × 6.5″ H) with a mesh lid. We used a 40 W incandescent heat bulb suspended above one end of the cage to maintain a temperature gradient from ∼25–40°C within cages, allowing for natural behavioral thermoregulation. Additionally, we placed a 39 W 10.0 UVB light tube horizontally across cages to provide a source of UV light. UV and ambient light were provided on a 14hr:10hr daily light: dark cycle with heat bulbs turned on in alternating 1 h on/1 h off intervals for a total of 6 h per day during the diurnal light cycle. Inside each cage, lizards were provided with a basking platform doubling as a shelter and a Petri dish filled with distilled water. We replaced the water and misted cages daily. We fed lizards every other day a diet of crickets and mealworms dusted with reptile vitamin supplement *ad libitum*. Between 14–16 June 2024, 2–3 fecal samples were opportunistically collected from three lizards. The three lizards were selected because they represent three distinct common wall lizard color morphs (orange, yellow, and white; Andrade et al. 2019). Individual fecal samples were removed from the cage using sterilized forceps and placed in sterile cryovials containing 1 ml of sterilized 10% glycerol in PBS. The samples were stored at −80°C until bacterial cultivation.

### Bacterial cultivation

Avian and lizard samples were processed independently using established laboratory protocols optimized at different institutions; consequently, some methodological details differ between host groups. Fecal samples and the negative field control were vigorously vortexed and serially diluted from 10^–1^ to 10^–6^. For aerobic cultivation of avian fecal samples, we used Brain Heart Infusion (BHI; non-selective) medium for cultivation of generalist taxa and “L9” (Yim et al. 2010) medium for selective cultivation of *Sphingomonas* spp. For aerobic cultivation of lizard fecal samples, we used BHI and Tryptic Soy Agar (TSA; non-selective). For each dilution, we prepared two replicate agar plates (100 × 15 mm) under a biosafety cabinet. On each plate, we spread 100 µl of diluted sample using 5–10 sterile glass beads (Zymo Cat. S1001). We included a negative (30% glycerol in PBS) and positive (*Sphingomonas paucimobilis*; ATCC 10829) control for each batch of samples processed, and we discarded batches of plates if negative controls had any growth. Plates were incubated aerobically at 30°C (lizard samples) or 35°C (avian samples) for 24–48 h or until sufficient growth occurred. We selected total cultures exhibiting distinct, well-isolated colonies of bacteria for phenotypic characterization of color, form, margin shape, texture, size, opacity, and elevation (Table S1). Colonies demonstrating distinct morphologies were then isolated by streaking on fresh agar plates. Once inoculated, we incubated the plates at 30°C or 35°C and monitored them every 24 h to prevent overgrowth. Any contaminated plates or plates that exhibited growth of more than one isolate were discarded.

For anaerobic cultivation (avian samples only), BHI plates were prepared and pre-reduced by placing them in an anaerobic container (Mitsubishi Gas Chemical) overnight. Samples were processed as previously described, but our positive control was *Fusobacterium nucleatum* subsp. *polymorphum* (ATCC 10953). Plates were immediately placed in anaerobic containers along with AnaeroPacks (Mitsubishi Gas Chemical) and an anaerobic indicator to create and confirm an oxygen-free environment. Containers were incubated at 35°C for 48–72 h. After incubation, unique colonies were phenotypically characterized, selected, and streaked onto fresh plates as previously described, but under anaerobic conditions.

We collected individual colony isolates from T-streak plates that were ∼0.5 cm^2^ in size with a sterile loop and incubated them in 1.5–2 ml of sterile broth to grow liquid cultures. We placed the samples (including a negative control containing only sterile broth) into an orbital incubator at 35°C at 120 r/m for 18–24 h under either aerobic or anaerobic conditions. We discarded liquid culture batches if any negative controls had growth. Half of the liquid culture was then mixed in 30% glycerol in PBS for long-term storage and the remaining half was centrifuged and decanted such that the pellets could be used as input for PCR and sequencing (below).

### 16S rRNA gene sequencing

For each isolate cultured from avian samples, we amplified the full-length 16S rRNA gene (including variable regions V1 to V9) by performing colony PCR amplification using 5 µM universal 16S primers 27F (5′-AGAGTTTGATCMTGGCTCAG-3′) and 1492R (5′-CGGTTACCTTGTTACGACTT-3′; Lane 1991). PCRs consisted of 6.5 µl nuclease-free water, 2 µl forward primer, 2 µl reverse primer, 12.5 µl LongAmp Hot Start*Taq* 2X Master Mix (New England BioLabs, M0533S), and ∼0.2 µl of pelleted bacterial cells (i.e. gently touching a pipette tip to the pellet). Reaction conditions were as follows: initial denaturation at 94°C for 30 s, followed by 30 cycles of 94°C for 30 s, 55°C for 40 s, 65°C for 90 s, followed by a final extension at 65°C for 10 min. PCR products were visualized on 1% agarose gels with SYBR Safe gel stain. Isolates that failed to amplify using colony PCR were regrown on plates and DNA was extracted with the ZymoBIOMICS DNA Miniprep Kit (D4300). PCRs were repeated under the same conditions using 2 µl of extracted DNA. We purified PCR products using Serapure beads (0.6x volume) and eluted in 25 µl of 10 µM Tris-HCl. The cleaned products were quantified with a Qubit 3.0 Fluorometer (Invitrogen) using the dsDNA HS Assay Kit (Q32854).

To prepare sequencing libraries, we used the Rapid Sequencing V14 Barcoding Kit (Oxford Nanopore Technologies [ONT], SQK-RBK114.98) following the manufacturer’s instructions (version RAA_9198_v114_revK_11Dec2024) with slight modifications. Specifically, we prepared half-size reactions (e.g. 25 ng of amplicon DNA in 4.5 µl of volume instead of 50 ng in 9 µl of volume) and doubled the incubation times (e.g. 20 min instead of 10 min). The barcoded and pooled libraries were loaded on a Flongle Flow Cell (ONT, FLO-FLG114) that was fitted to a MinION Mk1B device (ONT, MIN-101B) and sequenced. We used MinKNOW software (version 24.11.10) for raw data collection. We specified high accuracy basecalling and filtered reads to a minimum quality score of 9 and a minimum read length of 200 bp. We used the EPI2ME (desktop application version 5.2.3) “wf-amplicon” workflow (version 1.1.4) to trim adapters and generate *de novo* consensus sequences for each barcoded amplicon. We downsampled to 500 reads, specified a maximum read length of 2000 bp, and set the minimum number of reads of 150. Raw reads are deposited in the NCBI Short Read Archive (SRA) database (Table S2).

Samples that failed to meet these thresholds or failed during consensus sequence generation (n = 5) were alternatively sequenced (Sanger) in both directions by the Roswell Park Comprehensive Cancer Center DNA Sequencing Lab on an Applied Biosystems 3500 Genetic Analyzer (avian samples). All lizard samples (n = 5) were sequenced (Sanger) via direct colony sequencing on an Applied Biosystems 3730xl DNA Analyzer at Genewiz (Azenta). Forward and reverse sequences were assembled using the “isolateR” package (Daisley et al. 2024), specifying a sliding window cutoff of 20. Sanger sequences are deposited in GenBank (Table S2).

### Taxonomic classification, phylogenetic, and diversity analyses

We uploaded our consensus sequences to the Alignment, Classification and Tree Service on the SILVA website (https://www.arb-silva.de/aligner/), which uses SINA version 1.2.12 (Pruesse et al. 2012). We enabled the “search and classify” option to classify our sequences against the SILVA database; we specified a minimum identity of 95% and rejected sequences with <70% identity (Quast et al. 2013). For isolates that were not identified to the genus level with SILVA, we used the BLASTn program on the NCBI website (https://blast.ncbi.nlm.nih.gov/; Altschul et al. 1990) and classified the genus based on the most common genus of the top 50 hits as sorted by e-value.

We ensured all reads were oriented in the same direction before combining into a single FASTA for alignment. We performed multisequence alignment with MAFFT version 7.525 (Katoh and Standley 2013) using the default parameters (–auto). We included *Adhaeribacter pallidiroseus* (phylum Bacteroidota; NCBI accession NR_181905) as our outgroup to root the tree due to its ancestral placement from the ingroup taxa we identified. We estimated a maximum likelihood phylogeny using the alignment (1 541 bp) in IQ-Tree version 2.4.0 (Minh et al. 2020). We used 1000 ultrafast bootstrap replicates and 1000 SH-aLRT replicates as measures of branch support. The best model of nucleotide substitution was estimated in IQ-Tree prior to the analysis using ModelFinder (Kalyaanamoorthy et al. 2017) and corrected Akaike Information Criterion (AICc).

To define amplicon sequence variants (ASVs), we first pre-clustered sequences into operational taxonomic units (OTUs) at 99% identity using VSEARCH version 2.30.2 (“cluster_fast”), with minor manual adjustments based on monophyly. Then, sequences within each OTU were aligned using MAFFT, and leading and trailing gaps were removed to define a shared homologous region. Trimmed sequences were then dereplicated using VSEARCH (“fastx_uniques”) into unique ASVs.

To place our isolates into a broader microbiological context, we conducted a targeted literature search using the Web of Science database to identify experimentally supported reports of carotenoid-associated pigmentation (e.g. color in culture, chemical confirmation via chromatography) among representatives of each bacterial genus. We used quotation marks around each genus name plus the Boolean operator “AND” and the term “carotenoid*”. We screened up to 10 publications (articles) returned in order of relevance for evidence of carotenoid production. We excluded studies involving clonal transformations (e.g. bioengineering carotenoid biosynthesis genes into heterologous hosts) and those relying on genomic inference (e.g. putative carotenoid function based only on the presence of carotenoid biosynthesis genes), as these approaches do not demonstrate carotenoid production by naturally occurring isolates. Results were based upon taxonomy at the time of publication.

We tested whether microbiome community composition was structured by host species identity or sampling site using PERMANOVA. We generated a matrix of ASV presence/absence among samples and calculated unweighted UniFrac distance using the “distance” function in phyloseq version 1.50.0 (McMurdie and Holmes 2013). Unweighted UniFrac distance estimates phylogenetic dissimilarity among communities based on branch lengths in the ASV phylogeny. We conducted PERMANOVA tests on the unweighted UniFrac distance matrix using the “adonis2” function in vegan version 2.6–10 (Oksanen et al. 2025) by assessing the marginal effects of each term (by = “margin”). We also tested for differences in alpha diversity between species and sites by estimating Faith’s phylogenetic diversity (PD) in each sample using the “estimate_pd” function in btools version 0.0.1 (Battaglia 2019), fitting a linear model and running a Type II analysis-of-variance (ANOVA) using car version 3.1–3 (Fox and Weisberg 2019).

### Analysis of carotenoids

To assess whether bacterial pigments fell within the expected range of wavelengths at maximum absorbance for carotenoids, we performed an absorbance assay. To do so, we re-cultured isolates from stocks in sterile broth under either aerobic or anaerobic conditions to maximum density based on OD600 values. Samples were centrifuged into pellets and the supernatant was decanted. We added 800 µl of a methanol: acetone (7:3 V/V) solution to extract pigments from the bacterial pellets (Toomey et al. 2022). Samples were then either directly incubated at 60°C for 90 min at 250 r/m on a shaking incubator (avian samples) or were first sonicated using a sonicating wand for 20 s at 22 000 Hz then incubated at 60°C for 90 min at 400 r/m on a shaking incubator (lizard samples). A negative control containing only the methanol: acetone solution was also included. Following incubation (and once cells were visibly devoid of color), we centrifuged samples to pellet any cell debris. We then transferred 200 µl of the supernatant into a 96-well plate, including the negative control. We performed absorbance assays on a Tecan Spark multimode plate reader using the SparkControl program version 2.3 (avian samples) and on a Biotek Synergy H1 multimode plate reader using the Gen5 program version 1.11 (lizard samples). We measured absorbance from 300–700 nm (avian samples) or from 260–700 nm (lizard samples), taking a reading every 2 nm. Absorbance values were standardized relative to the negative control in each plate.

We considered absorbance spectra to be consistent with a carotenoid if the wavelength at maximum absorbance (lambda max) fell between 378 nm and 512 nm (Rodriguez-Amaya 2001). To identify lambda max, we used the function “find_peaks” in the R package photobiology version 0.14.0 (Aphalo 2015) to detect lambda values larger than five consecutive values on either side (span = 11, global.threshold = 0.5). Three researchers (SG, VG, KS) independently inspected the spectra for validation (Fig. S1). If two or more observers disagreed with the results of “find_peaks” for a certain sample, we considered that spectrum as “missing data” as it likely represents either a false positive or false negative.

## Results

### Phenotypic diversity

We cultured a total of 152 morphologically distinct bacterial isolates across 20 individual birds and five distinct isolates across three individual lizards (total n = 157; Table 1). *Sphingomonas paucimobilis* grew on our selective (“L9”) media positive control in aerobic conditions and *Fusobacterium nucleatum* subsp. *polymorphum* grew on our non-selective media in anaerobic conditions. No bacteria grew on our negative field control in either aerobic or anaerobic conditions.

**Table 1:** Number of phenotypically unique isolates by host species and media type.

Host species	N	Aerobic		Anaerobic
		Non-selective	Selective	Non-selective
Purple Martin	4	24	3	12
Blue-winged Warbler	5	22	0	9
Common Yellowthroat	3	11	0	6
Chestnut-sided Warbler	3	14	1	9
Gray Catbird	2	8	3	5
Hooded Warbler	1	6	1	4
Yellow Warbler	2	10	0	4
Common Wall Lizard	3	5	0	N/A

N represents the number of host individuals.

**Figure 1:**
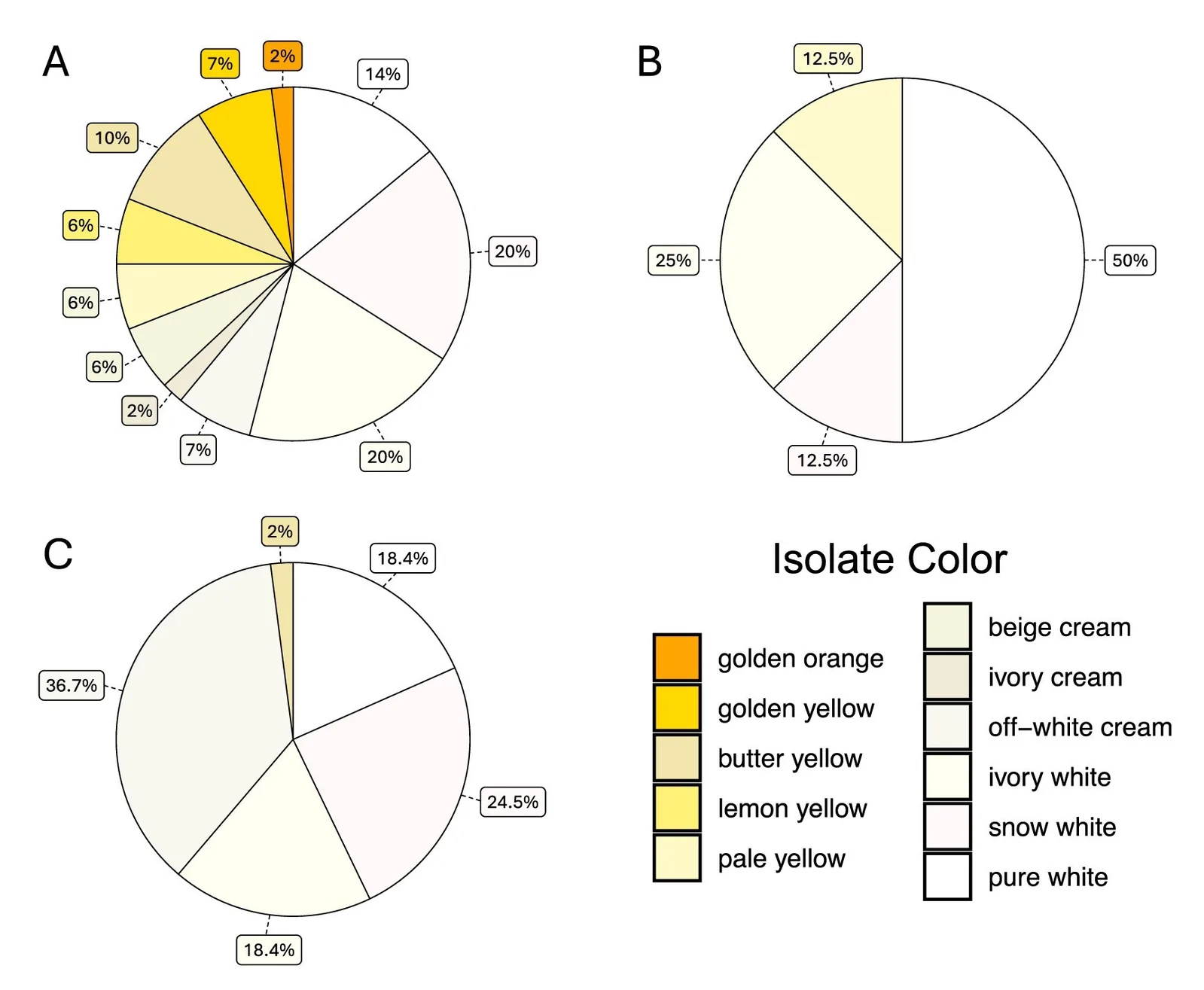
Distribution of color phenotypes observed in 157 isolates cultured from 20 individual birds and three lizards on (A) non-selective aerobic, (B) selective aerobic, and (C) non-selective anaerobic media.

We characterized initial colony phenotype upon growing total cultures, but herein we report only their phenotype in isolation (i.e. after T-streaking), as we occasionally observed minor differences. Across both anaerobic and aerobic conditions and both non-selective and selective media, 78.9% of isolates were shades of white or cream (Table S1). This pattern was generally consistent across oxygen conditions and media as well, such that ivory white and snow white were the most frequent colors for non-selective aerobic media (Fig. 1A), pure white was the most frequent color for selective aerobic media (Fig. 1B), and off-white cream was the most frequent color for non-selective anaerobic media (Fig. 1C). We observed four distinct shades of yellow and one shade of orange across isolates (Table S1; Fig. 1). There were 33 isolates in total (21%) that expressed either shades of yellow or orange, which is consistent with the presence of carotenoids, and represents a similar color phenotype to the majority of congeneric bacteria identified from our literature search (Table 2).

**Table 2:** Summary of isolate taxonomy, phenotypes, and literature-derived evidence of carotenoid production among congeneric taxa.

Phylum	Class	Order	Family	Genus	n OTUs	n (%) isolates	n (%) carotenoid (color)	n (%) carotenoid (UV-Vis)	Citation	Species	Source	Color in culture	Pigment chemically confirmed
Actinomycetota	Actinobacteria	Micrococcales	Microbacteriaceae	Plantibacter	1	1 (0.68%)	1 (100%)	1 (100%)	No results				
Actinomycetota	Actinobacteria	Micrococcales	Micrococcaceae	Micrococcus	1	1 (0.68%)	1 (100%)	1 (100%)	Thirkell 1969	Micrococcus radiodurans	not described	yellow, orange, red	yes
									Schwartzel and Cooney 1972	Micrococcus roseus	not described	not described	yes
Actinomycetota	Actinobacteria	Micrococcales	Micrococcaceae	Rothia	2	3 (2.04%)	0 (0%)	1 (33.33%)	No relevant results				
Actinomycetota	Actinobacteria	Mycobacteriales	Tsukamurellaceae	Tsukamurella	1	2 (1.36%)	2 (100%)	1 (50%)	No results				
Bacillota	Bacilli	Bacillales	Bacillaceae	Bacillus	2	2 (1.36%)	0 (0%)	0 (0%)	Khaneja et al. 2010	multiple Bacillus spp.	soil, sea water, and human gut	yellow, orange, pink	yes
Bacillota	Bacilli	Bacillales	Bacillaceae	Priestia	1	1 (0.68%)	0 (0%)	0 (0%)	No relevant results				
Bacillota	Bacilli	Bacillales	Caryophanaceae	Kurthia	1	1 (0.68%)	1 (100%)	1 (100%)	Koyanagi et al. 2021	Kurthia gibsonii	dragonfly feces	yellow	yes
Bacillota	Bacilli	Bacillales	Planococcaceae	Lysinibacillus	1	1 (0.68%)	1 (100%)	0 (0%)	No relevant results				
Bacillota	Bacilli	Exiguobacterales	Exiguobacteraceae	Exiguobacterium	1	1 (0.68%)	1 (100%)	1 (100%)	White et al. 2019	Exiguobacterium chiriqhucha strain RW2	microbialite	orange	no
									Sasidharan et al. 2013	Exiguobacterium aurantiacum; Exiguobacterium profundum	soil and air	yellow	yes
									Flora et al. 2026	Exiguobacterium arabatum; Exiguobacterium qingdaonense	marine sediment	orange	yes
									Petrovskaya et al. 2025	Exiguobacterium sibiricum	not described	yellow-orange	yes
									Takatani et al. 2025	Exiguobacterium sp. strain oki7	seaweed	orange	yes
Bacillota	Bacilli	Lactobacillales	Carnobacteriaceae	Carnobacterium	2	3 (2.04%)	0 (0%)	0 (0%)	No relevant results				
Bacillota	Bacilli	Lactobacillales	Enterococcaceae	Enterococcus	8	31 (21.09%)	5 (16.13%)	15 (48.39%)	Tyrrell et al. 2002	Enterococcus gilvus and Enterococcus pallens	human gut clinical isolate	yellow	no
									Hagi et al. 2017	Enterococcus gilvus	not described	not described	yes
									Breithaupt et al. 2001	Enterococcus sulfureus; Enterococcus mundtii	vegetables; soil	not described	yes
									Hagi et al. 2014	Enterococcus gilvus	raw milk	yellow	yes
Bacillota	Bacilli	Lactobacillales	Streptococcaceae	Lactococcus	2	29 (19.73%)	0 (0%)	5 (17.24%)	No relevant results				
Bacillota	Bacilli	Lactobacillales	Streptococcaceae	Streptococcus	1	1 (0.68%)	0 (0%)	0 (0%)	Taylor and Davies 1976	Streptococcus faecium	not described	not described	yes
									Taylor and Davies 1974a	Streptococcus faecium	not described	not described	yes
									Taylor and Davies 1974b	Streptococcus faecium	soil	yellow	yes
									Whiteley and D'Souza 1989	Streptococcus faecium	lunch meat	yellow	yes
Bacillota	Bacilli	Staphylococcales	Staphylococcaceae	Mammaliicoccus	1	2 (1.36%)	0 (0%)	1 (50%)	No results				
Bacillota	Bacilli	Staphylococcales	Staphylococcaceae	Staphylococcus	1	2 (1.36%)	0 (0%)	0 (0%)	Douglas and Parisi 1966	Staphylococcus aureus	not described	yellow	yes
Bacteroidota	Bacteroidia	Flavobacteriales	Weeksellaceae	Apibacter	1	3 (2.04%)	3 (100%)	2 (66.67%)	No results				
Pseudomonadota	Alphaproteobacteria	Hyphomicrobiales	Rhizobiaceae	Agrobacterium	1	1 (0.68%)	0 (0%)	0 (0%)	Yokoyama et al. 1994	Agrobacterium aurantiacum	sea water	yellow, orange, red	yes
									Yokoyama and Miki 1995	Agrobacterium aurantiacum	sea water	not described	yes
									Yokoyama et al. 1995	Agrobacterium aurantiacum	sea water	not described	yes
Pseudomonadota	Alphaproteobacteria	Hyphomicrobiales	Rhizobiaceae	Brucella	1	1 (0.68%)	0 (0%)	0 (0%)	No relevant results				
Pseudomonadota	Gammaproteobacteria	Burkholderiales	Comamonadaceae	Delftia	1	1 (0.68%)	0 (0%)	0 (0%)	No results				
Pseudomonadota	Gammaproteobacteria	Enterobacterales	Enterobacteriaceae	Cedecea	1	1 (0.68%)	0 (0%)	0 (0%)	No relevant results				
Pseudomonadota	Gammaproteobacteria	Enterobacterales	Enterobacteriaceae	Enterobacter	2	4 (2.72%)	1 (25%)	2 (50%)	Lehner et al. 2006	Enterobacter sakazaki	human clinical isolate	yellow	yes
Pseudomonadota	Gammaproteobacteria	Enterobacterales	Enterobacteriaceae	Klebsiella	1	3 (2.04%)	0 (0%)	0 (0%)	No relevant results				
Pseudomonadota	Gammaproteobacteria	Enterobacterales	Enterobacteriaceae	Kluyvera	1	1 (0.68%)	0 (0%)	0 (0%)	No results				
Pseudomonadota	Gammaproteobacteria	Enterobacterales	Enterobacteriaceae	Kosakonia	1	1 (0.68%)	1 (100%)	1 (100%)	Bateman et al. 2025	Kosakonia sp.	musk melon	yellow-orange	no
Pseudomonadota	Gammaproteobacteria	Enterobacterales	Enterobacteriaceae	Raoultella	1	1 (0.68%)	0 (0%)	0 (0%)	No relevant results				
Pseudomonadota	Gammaproteobacteria	Enterobacterales	Erwiniaceae	Erwinia	1	4 (2.72%)	0 (0%)	0 (0%)	Rezzonico et al. 2016	Erwinia gerundensis	fruit	yellow	no
									Hundle et al. 1991	Erwinia herbicola	ATCC 39 368	not described	yes
Pseudomonadota	Gammaproteobacteria	Enterobacterales	Erwiniaceae	Pantoea	2	15 (10.2%)	14 (93.33%)	14 (93.33%)	Bateman et al. 2025	multiple Pantoea spp.	plants, insects, soil, gastropods, humans	yellow, orange, red	no
									Gallego et al. 2026	Pantoea sp.	bird feces	yellow	no
Pseudomonadota	Gammaproteobacteria	Enterobacterales	Hafniaceae	Hafnia-Obesumbacterium	1	2 (1.36%)	0 (0%)	1 (50%)	No relevant results				
Pseudomonadota	Gammaproteobacteria	Enterobacterales	Morganellaceae	Morganella	2	2 (1.36%)	0 (0%)	1 (50%)	No relevant results				
Pseudomonadota	Gammaproteobacteria	Enterobacterales	Morganellaceae	Providencia	1	1 (0.68%)	0 (0%)	0 (0%)	No relevant results				
Pseudomonadota	Gammaproteobacteria	Enterobacterales	Orbaceae	Orbus	1	1 (0.68%)	0 (0%)	0 (0%)	No results				
Pseudomonadota	Gammaproteobacteria	Enterobacterales	Yersiniaceae	Rahnella	1	3 (2.04%)	0 (0%)	2 (66.67%)	No relevant results				
Pseudomonadota	Gammaproteobacteria	Enterobacterales	Yersiniaceae	Serratia	6	18 (12.24%)	1 (5.56%)	6 (33.33%)	Stancu 2016	Serratia marcescens IBBPo15	oily sludge	not described	yes
									Courington and Goodwin 1955	Serratia marinorubra	not described	purple	no
Pseudomonadota	Gammaproteobacteria	Lysobacterales	Lysobacteraceae	Stenotrophomonas	1	2 (1.36%)	1 (50%)	1 (50%)	Yi et al. 2010	Stenotrophomonas panacihumi	soil	yellow	no
Pseudomonadota	Gammaproteobacteria	Pseudomonadales	Pseudomonadaceae	Pseudomonas	1	1 (0.68%)	0 (0%)	1 (100%)	Fukaya et al. 2018	Pseudomonas sp.	dragonfly feces	yellow	yes
									Senocak et al. 2026	Pseudomonas gebzensis	surface of solar panel	yellow	no
									Peel and Quayle 1961	Pseudomonas AM1	air	pink	yes
Unknown	Unknown	Unknown	Unknown	Unknown	na	10 (6.8%)	0 (0%)	4 (40%)					

The first five columns represent isolate taxonomy, including phylum, class, order, family, and genus. The number of OTUs per genus is indicated in the “n OTU” column. The number and percentage of isolates per genus (out of 147 taxonomically identified) are indicated in the “n (%) isolates” column. The number and percentage of isolates within each genus classified as carotenoid-positive based on color phenotype in culture are indicated in the “n (%) carotenoid (color)” column, and those classified as carotenoid positive by UV-Vis spectral analysis are indicated in the “n (%) carotenoid (UV-Vis)” column. The final five columns summarize results from our literature search of congeneric taxa, including citation(s), species identity, isolate source, reported color in culture (if available), and whether the pigment was chemically confirmed as a carotenoid. Genera listed with “no relevant results” indicate that no publications meeting our search criteria provided evidence consistent with carotenoid production, whereas those listed with “no results” indicate that no articles were returned by the search.

We further characterized isolates based on their overall shape (“form”), relative size, edge (“margin”) texture, opacity, and elevation (relative height). The most common shape of isolates was circular (78.9% in total; 75.9% of aerobic colonies, 85.7% of anaerobic colonies), with the majority having a surface texture being described as moist and shiny (84.7% in total; 84.3% of aerobic colonies, 85.7% of anaerobic colonies). The variation in size was more evenly distributed among aerobic colonies than anaerobic isolates: 27.7% aerobic isolates were described as tiny, 32.4% were described as small, 30.5% as medium, whereas 77.6% of anaerobic isolates were described as small. The edge texture of colonies was primarily smooth (77.7% in total; 79.6% of aerobic colonies, 73.5% of anaerobic colonies). Most isolates grown in aerobic conditions were either opaque (53.7%) or translucent (40.7%), and a similar pattern was observed for anaerobic isolates (38.8% opaque; 42.9% translucent). Most isolates were flat (64.9% in total; 69.4% of aerobic, 55.1% of anaerobic), but they were also often described as umbonate (i.e. having a slightly raised bump in the center of the colony; 21.7% in total; 13.9% of aerobic, 38.8% of anaerobic). All phenotype data can be found in Table S2.

**Figure 2:**
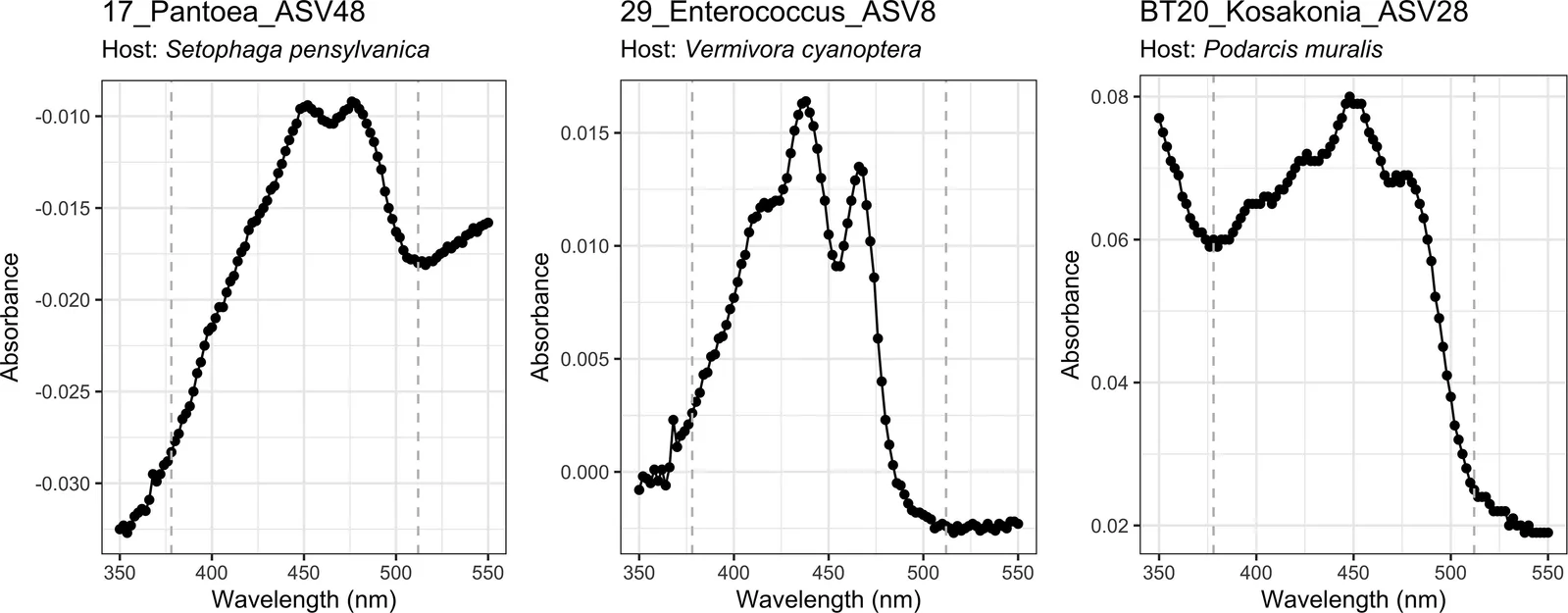
Example absorbance spectra that are consistent with the presence of carotenoids; dashed gray lines represent the lower (378 nm) and upper (512 nm) bounds of carotenoid wavelengths.

**Figure 3:**
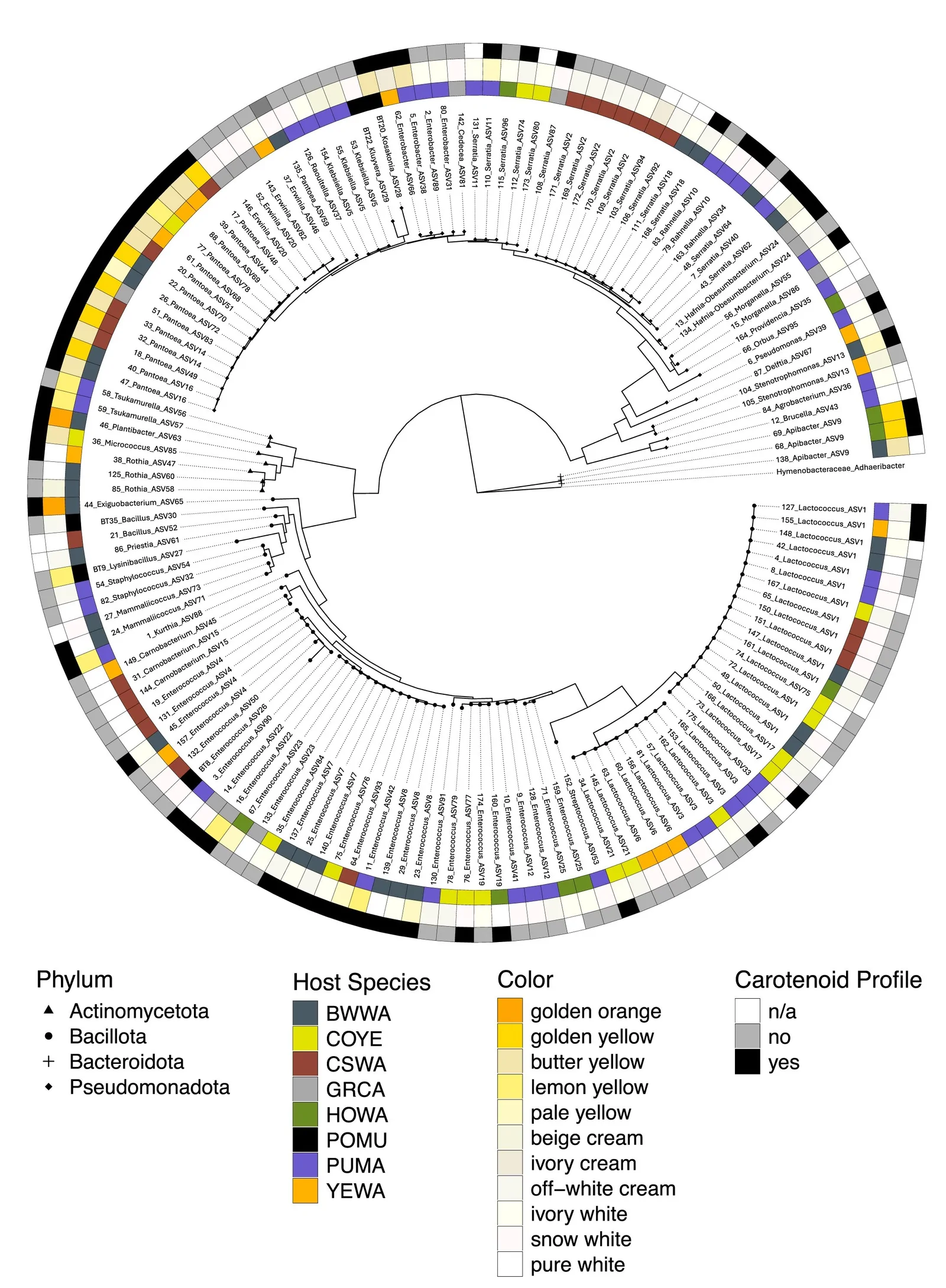
Maximum likelihood phylogenetic tree estimated from the 16S rRNA gene of 147 bacterial isolates cultured from wild sauropsid fecal samples. Tips are labeled with the genus and ASV of each isolate, and shapes at the end of tips represent their taxonomic classification at the level of phylum (*Actinomycetota*—triangle; *Bacillota*—circle; *Bacteroidota*—cross; *Pseudomonadota*—diamond). The innermost ring represents the host species from which the isolate was isolated (Blue-winged Warbler, BWWA—slate blue; Common Yellowthroat, COYE—yellow; Chestnut-sided Warbler, CSWA—chestnut red; Gray Catbird, GRCA—light gray; Hooded Warbler, HOWA—olive green; Common Wall Lizard, POMU—black; Purple Martin, PUMA—purple; Yellow Warbler, YEWA—orange); the middle ring represents the color of the isolate; the outermost ring represents whether the isolate is consistent (black) or inconsistent (gray) with a carotenoid based on absorbance spectra, or could not be determined (white).

We performed absorbance assays on 156 of the 157 morphologically distinct bacterial isolates (one isolate failed to regrow for this assay). Lambda max values fell within the expected range for carotenoids for 62 isolates (39.7%; Fig. 2). These isolates belong to 19 genera, with *Pantoea* and *Enterococcus* most frequently represented (Fig. 3; Table 2). All eleven streak colors were represented among these 62 isolates, with 29 (46.8%) being a shade of yellow or orange (Table S2). The majority of these 62 isolates were cultivated under aerobic conditions (82.3%). There were 74 isolates (47.4%) with lambda max values that fell outside of the expected carotenoid range, representing 21 genera (Table S2). *Lactococcus* and *Enterococcus* were the two most prevalent genera in this group. Streak colors were generally shades of white and cream, although two lemon yellow isolates and one pale yellow isolate were included with this group (Table S2). The cultivation conditions were more evenly split for isolates whose lambda max values fell outside of the expected range for carotenoids (58.1% aerobic, 41.9% anaerobic). We could not determine lambda max for 20 isolates (12.8%).

### Genotypic diversity

Of our 157 isolates, we successfully obtained sequencing data from 147 (Fig. 2; Table S2). Of the 147 successful samples, the average number of Nanopore FASTQ reads that passed our filters was 1616.8 (± 111 [SE]), with a minimum of 244 reads and a maximum of 6435 reads.

Our 147 isolates are representative of four phyla, with the two most common being *Bacillota* (50.3%) and *Pseudomonadota* (42.8%). These were further subdivided into five classes, with the two most common being *Bacilli* (50.3%) and *Gammaproteobacteria* (41.5%). Our isolates belong to 12 orders (the most common being *Lactobacillales* [43.5%] and *Enterobacterales* [38.7%]) that encompass 22 families. The most common families include *Enterococcaceae* and *Streptococcaceae* (both representing ∼20% of isolates), *Yersiniaceae* (14.8%), and *Erwiniaceae* (12.9%). Our samples encompass 35 different genera, with the most common being *Enterococcus* (21.1%), *Lactococcus* (19.7%), *Serratia* (12.2%), and *Pantoea* (10.2%). Genera that were unique to aerobic conditions (n = 21) include *Agrobacterium, Bacillus, Brucella, Delftia, Enterobacter, Exiguobacterium, Kluyvera, Kosakonia, Kurthia, Lysinibacillus, Mammaliicoccus, Micrococcus, Morganella, Orbus, Plantibacter, Priestia, Pseudomonas, Rothia, Staphylococcus, Stenotrophomonas*, and *Tsukamurella*. Isolates that belong to the genus *Pantoea* were almost exclusively grown under aerobic conditions, but one isolate grew under anaerobic conditions. Genera that were unique to anaerobic conditions (n = 4) included *Cedecea, Providencia, Raoultella*, and *Streptococcus*. Genera that grew under both aerobic and anaerobic conditions (n = 10) included *Apibacter, Carnobacterium, Enterococcus, Erwinia, Hafnia-Obesumbacterium, Klebsiella, Lactococcus, Pantoea, Rahnella*, and *Serratia*. No *Sphingomonas* spp. were recovered on either selective or non-selective media.

All host species except Chestnut-sided Warbler had at least one unique bacterial genus. These included *Agrobacterium, Brucella, Klebsiella, Kurthia, Providencia, Pseudomonas, Rahnella, Raoultella, Staphylococcus, Streptococcus*, and *Tsukamurella*, which were unique to Purple Martins; *Exiguobacterium, Mammaliicoccus, Plantibacter*, and *Priestia* were unique to Blue-winged Warblers; *Cedecea* and *Hafnia-Obesumbacterium* were unique to Gray Catbirds; *Delftia* was unique to Yellow Warblers; *Micrococcus* was unique to Common Yellowthroats; *Orbus* was unique to Hooded Warblers; and *Kluyvera, Kosakonia*, and *Lysinibacillus* were unique to Common Wall Lizards.

We further subdivided the 35 genera into 96 unique ASVs. Most genera were placed into one or two ASVs and three were divided into more than ten ASVs (*Enterococcus* = 19 ASVs; *Pantoea* = 13 ASVs; *Serratia* = 12 ASVs). Most ASVs were unique to a single host species (92.7%). No ASVs were shared by all eight host species, but ASV1 (*Lactococcus*) was shared by six, including Blue-winged Warbler, Common Yellowthroat, Chestnut-sided Warbler, Hooded Warbler, Yellow Warbler, and Purple Martin.

Our phylogeny was highly supported, primarily at deeper nodes, but shallower nodes also had high bootstrap and SH-aLRT support (Fig. S2). According to SILVA taxonomy, several genera were not monophyletic. For example, *Enterococcus* and *Serratia* were polyphyletic, such that their most recent common ancestor encompasses isolates assigned to multiple other genera. *Enterobacter* and *Pantoea*, though they each formed a largely monophyletic clade, exclude one descendent lineage, indicating instances of paraphyly.

**Figure 4:**
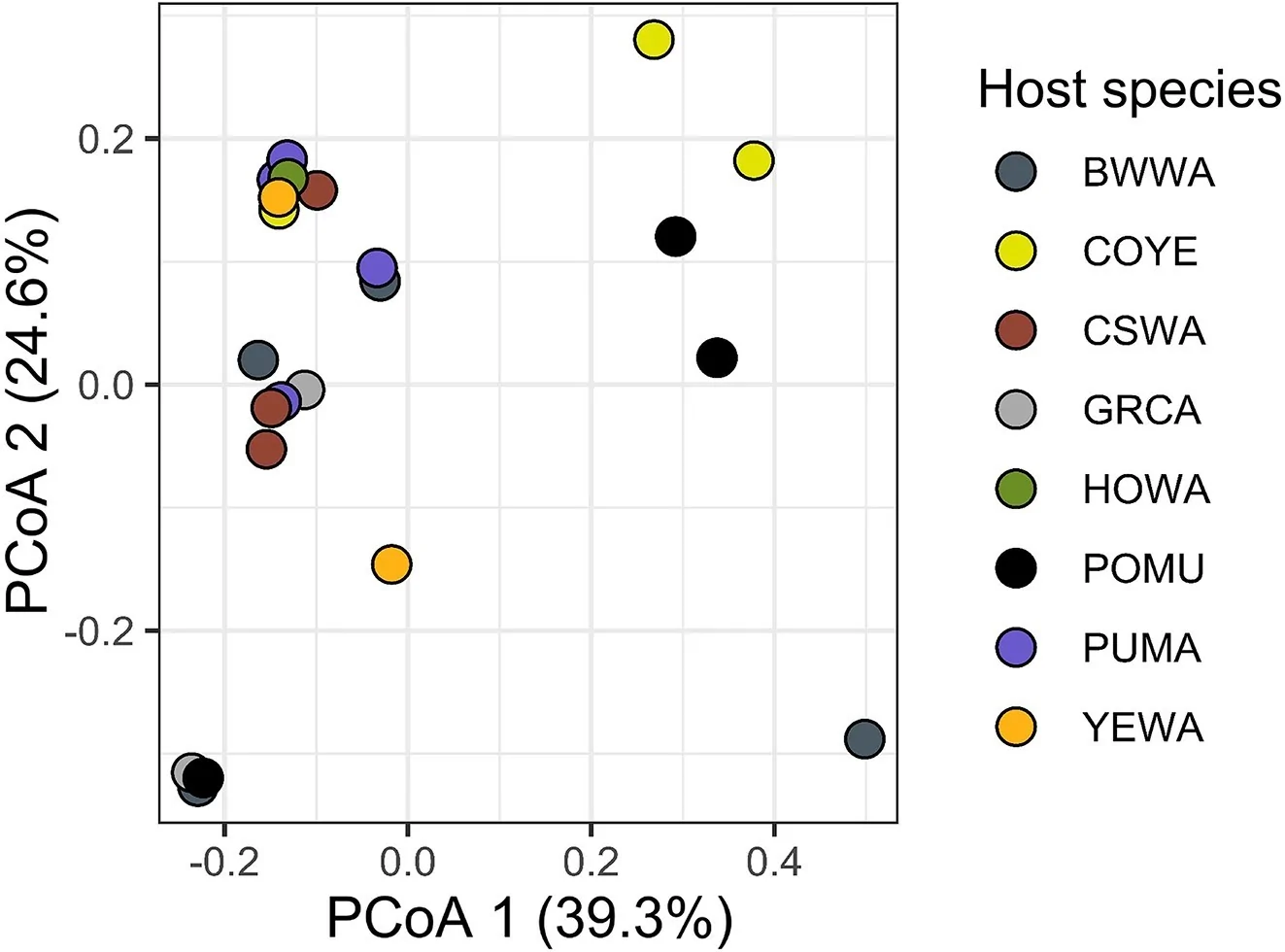
Principal coordinates analysis (PCoA) of bacterial isolates among samples based on ASV presence/absence using unweighted UniFrac distances. Each point represents a sample, and colors indicate host species by their species code (Blue-winged Warbler, BWWA—slate blue; Common Yellowthroat, COYE—yellow; Chestnut-sided Warbler, CSWA—chestnut red; Gray Catbird, GRCA—light gray; Hooded Warbler, HOWA—olive green; Common Wall Lizard, POMU—black; Purple Martin, PUMA—purple; Yellow Warbler, YEWA—orange).

**Figure 5:**
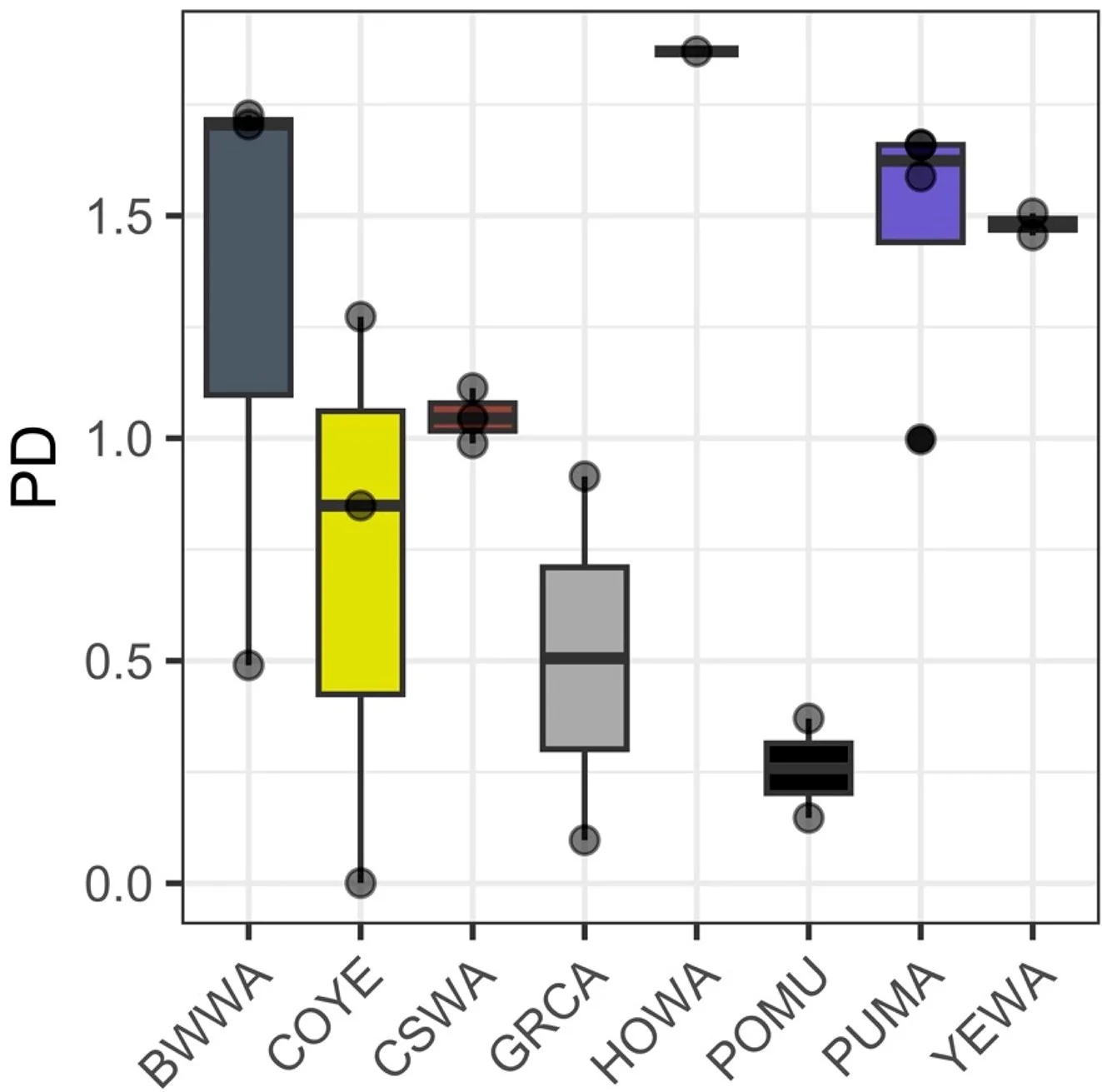
Faith’s phylogenetic diversity (PD) alpha index across host species. Each point represents an individual host and host species are indicated by their species code and associated color (Blue-winged Warbler, BWWA—slate blue; Common Yellowthroat, COYE—yellow; Chestnut-sided Warbler, CSWA—chestnut red; Gray Catbird, GRCA—light gray; Hooded Warbler, HOWA—olive green; Common Wall Lizard, POMU—black; Purple Martin, PUMA—purple; Yellow Warbler, YEWA—orange).

PERMANVOA tests indicated that neither host species (R^2^ = 0.2, *P* = 0.14) nor sampling site (R^2^ = 0.1, *P* = 0.40) explained any variation between microbiomes (Fig. 4). Phylogenetic diversity differed significantly across species (F = 4.33, *P* = 0.03)—warblers and Purple Martins tended to have higher values than Gray Catbirds and Common Wall Lizards—but did not differ among sampling sites (F = 3.04, *P* = 0.09; Fig. 5).

## Discussion

Our results provide the first empirical evidence that gut-associated bacteria isolated from wild sauropsids exhibit diverse pigment phenotypes that are consistent with carotenoids, including several genera for which carotenoid production has not yet been reported. Broad sampling and high-resolution sequencing demonstrate that these carotenoid-producing bacteria are not restricted to a single taxonomic group, sampling site, or host group (species, family, or class), which suggests that they may represent a ubiquitous, yet hidden, component of the sauropsid gut microbiome. Furthermore, carotenoid-producing bacteria were recovered from hosts with and without conspicuous carotenoid-based coloration, suggesting that the presence of carotenoid-producing gut bacteria may contribute to not only host pigmentation, but also serve other functions through their impact on host physiology. Together, these findings provide support for the view that microbial symbionts may be a valuable source of carotenoids to their host.

Although the majority of the isolates were non-pigmented, we frequently observed yellow- or orange-pigmented isolates (Fig. 1). Every host species had at least one pigmented isolate, and these phenotypes spanned all four bacterial phyla represented in our dataset, which is consistent with comparative genomic studies demonstrating that carotenoid biosynthetic proteins are widely distributed across bacterial lineages (Klassen 2010). This widespread occurrence across distantly related bacterial and host lineages suggests that carotenoid-like pigments are a recurrent trait in gut-associated bacteria rather than a taxonomic or host-specific anomaly. The yellow phenotype was most often associated with *Pantoea*, which was found only in our avian samples. All but one *Pantoea* were yellow pigmented and exhibited lambda max values within the expected range for carotenoids (Figs. 2 and 3, Table 2). *Pantoea* is known to synthesize carotenoids such as zeaxanthin (Misawa et al. 1990, Hundle et al. 1994), which suggests that the colorful pigments we observed likely reflect genuine functional potential for carotenoid biosynthesis. This interpretation is consistent with the presence of multiple carotenoid biosynthesis genes (e.g. *crt* genes) in a *Pantoea* genome assembly from these samples (“22_Pantoea_ASV70”; Gallego et al. 2026). Notably, *Pantoea* is also among the most prevalent taxa recovered from 16S metabarcoding across a broad sampling of warbler gut microbiomes (Baiz et al. 2023), and was prevalent among isolates from gray-breasted parakeet cloacal swabs (Beleza et al. 2021). Thus, despite our (avian) cultures relying on only two media types under two oxygen conditions (an approach that underrepresents unculturable or fastidious taxa) at a single incubation temperature (which was lower than typical avian body temperature), and with isolate selection based on colony morphology during the isolation process, we were still able to isolate some of the most dominant bacterial taxa that are known to be associated with the avian gut microbiome.

The recovery of carotenoid-producing microbiota from both avian and lizard samples in culture, despite minor differences in sample collection and processing protocols, suggests that these findings are robust to methodological approaches and supports the conclusion that these bacteria are widespread among sauropsids. A literature search of the genera cultured in this study revealed that at least five genera isolated from the guts of mammals and insects (*Bacillus, Kurthia, Enterococcus, Pantoea*, and *Pseudomonas*) have also been reported to exhibit evidence of carotenoid production (Table 2), which suggests that carotenoid production could be a broadly distributed trait among animal gut-associated bacteria. Notably, ten genera for which we observed evidence of carotenoid-like production lacked previous reports of such function (*Plantibacter, Rothia, Tsukamurella, Lysinibacillus, Lactococcus, Mammaliicoccus, Apibacter, Hafnia*-*Obesumbacterium, Morganella, Rahnella*; Table 2). This finding greatly expands the known taxonomic diversity of putative carotenoid-producing gut bacteria. Altogether, these results indicate that our culture-based sampling captured at least a subset (albeit an underrepresentation) of ecologically important and naturally abundant carotenoid-producing bacteria from sauropsid gut microbiomes.

In contrast to the relatively tight correlation between phenotype and genotype observed in *Pantoea, Enterococcus* and *Serratia* exhibited more heterogeneity under both aerobic and anaerobic conditions. These two genera were among the most prevalent taxa in our sampling (at least of avian hosts), which is consistent with a previous study of warbler microbiomes which found *Enterococcus* and an unidentified genus within the family *Yersiniaceae* (of which *Serratia* is a member) as the two most prevalent taxa (Baiz et al. 2023). *Enterococcus* was the most ASV-rich genus in our dataset (19 ASVs across 31 isolates), and *Serratia* was among the top three most ASV-rich (12 ASVs across 18 isolates; *Pantoea* exhibited 13 ASVs across 15 isolates). Both *Enterococcus* and *Serratia* produced isolates that fell within and outside of the expected carotenoid lambda max range and encompassed a spectrum of pigmented and non-pigmented color phenotypes (Fig. 3, Table 2). This pattern may suggest considerable genomic variation in the prevalence of carotenoid biosynthesis genes in these genera, a trend documented in other bacterial taxa. For example, the distribution of *crtN* and *crtM* genes (which encode for C30 carotenoid biosynthesis) has been found to be widely scattered across taxa within the bacterial family *Lactobacillaceae* and may be related to differential habitat adaptations (Eilers et al. 2024). An alternate possibility may be related to context-specific metabolic expression patterns: because isolates were grown under both aerobic and anaerobic conditions, shifts in pigment production may reflect facultative physiological responses (Pedraz et al. 2020), as have been documented in *Enterococcus* (Hagi et al. 2014) and *Serratia* (Rodríguez et al. 2023). Targeted genomic screening for carotenoid biosynthesis loci and controlled experiments manipulating oxygen availability (among other external factors) in these taxa would help to disentangle these possible mechanisms. Taken together, these results highlight that carotenoid-associated traits can vary widely even within bacterial genera, emphasizing the need to consider both genomic and environmental sources of functional diversity in host-associated microbiota.

The confirmed presence of carotenoid-producing bacteria expressing carotenoid-like phenotypes isolated from a diversity of sauropsid hosts adds novel context to the growing number of studies that have found correlations between host microbiomes and variation in phenotype (Nguyen et al. 2020, Marques Silva et al. 2023, Baiz et al. 2024, Koellsch et al. 2024, Slevin et al. 2025). More specifically, these findings raise the possibility that microbial pigment production in the gut could represent a previously unrecognized contributor to host coloration. Accordingly, three of the four genera most frequently represented in our isolates (*Pantoea, Enterococcus*, and *Serratia*) are also among the genera most commonly detected on wild bird feathers (Giorgio et al. 2018, Silva et al. 2022, Tran et al. 2022). The taxonomic overlap between internal gut-associated microbes and external integument-associated microbes supports the possibility of a functional link between carotenoid-producing bacteria in the gut and carotenoid signatures detected in the integument, either directly or indirectly. Direct paired comparisons of carotenoids produced by gut-associated bacteria and those deposited in host integuments (e.g. feathers, scales) are still lacking but represent an important next step in this field. Such studies would help determine whether microbial and host carotenoid pigments overlap and whether particular bacterial taxa or microbial carotenoid profiles are associated with differences in host pigmentation, and ultimately the functional consequences of those differences. Further experimental tests like providing antioxidant supplementations to hosts (Martínez-Renau et al. 2024) or inoculating germ-free hosts with carotenoid-producing bacteria could provide further evidence for causal links between the microbiome and measures of host pigmentation. In a natural experiment involving hybridizing birds, predictive metagenomics analyses revealed that carotenoid-associated plumage scores in *Vermivora* (Parulidae) hybrids were correlated with the predicted abundance of KEGG orthologs and enzymes in carotenoid biosynthesis pathways (Baiz et al. 2024). These findings also suggest that further research is warranted into the contributions of microbial carotenoids to host-associated processes beyond coloration (e.g. immunity and physiology). More broadly, the ecological or evolutionary mechanisms that enable carotenoid-producing bacteria (and generally any microbe) to persist in the host gut, and the conditions under which they might influence host traits, remain largely unknown (Decaestecker et al. 2024).

We observed a similar prevalence of carotenoid-producing bacteria in both avian and lizard hosts (∼40%), vertebrate lineages that diverged ∼280 million years ago (Kumar et al. 2017). This may suggest a deep evolutionary history of symbiotic associations between carotenoid-synthesizing bacteria and sauropsid hosts, and several factors may explain the prevalence of these bacteria in the sauropsid gut microbiome. First, because carotenoids protect bacteria from environmental stressors, they tend to flexibly inhabit and thrive under various conditions without strict niche specialization (Martino et al. 2016, Eilers et al. 2024). In fact, multiple genera recovered in this study have also been reported to exhibit evidence of carotenoid production in non-host environments including soil, air, and water (Table 2). The ability of these bacteria to persist across diverse non-host environments may increase opportunities for repeated dispersal and, subsequently, colonization of animal-associated niches (e.g. gut microbiomes). Consistent with this view, we isolated bacteria that expressed carotenoid-like phenotypes from every host species sampled at each of our study sites, and neither site nor host species were strong predictors of microbiome composition (Fig. 4). Similarly, many of these taxa may be common symbionts of prey, like *Pantoea* which is commonly associated with insects (Walterson and Stavrinides 2015), providing a plausible route of acquisition and explain its ubiquity among our samples. Alternatively, and regardless of their baseline environmental prevalence, hosts may selectively filter for carotenoid-synthesizing bacteria. Filtering could occur indirectly by some physiological mechanism and allow for the proliferation of carotenoid-synthesizing taxa over others (e.g. gut pH, temperature) or more directly if natural selection favors hosts that harbor high abundances of carotenoid-synthesizing bacteria. Carotenoids are a highly valuable resource for animals as they contribute to coloration, immune function, and oxidative stress protection (Blount 2004, Fiedor and Burda 2014, Toews et al. 2017), and if hosts are able to take advantage of carotenoids synthesized by gut microbes in addition to dietary sources, we would expect selection to favor the maintenance of such a mutualism. However, carotenoid expression may be condition dependent (Hagi et al. 2014), and further research is necessary to determine whether the bacteria we isolated here synthesize carotenoids within the host gut (i.e. not just in cultured isolation) and, if so, what functional role they may play.

Despite their potential functional importance, virtually nothing has been documented about the prevalence or diversity of carotenoid-producing gut bacteria in wild hosts. Our results suggest that some of the most prevalent bacterial taxa in wild sauropsid gut microbiomes are capable of producing carotenoids and are taxonomically widespread. These findings raise the possibility that the host gut microbiome serves as an underappreciated reservoir of biologically relevant and valuable carotenoids, although future work is needed to confirm the chemical identity of these compounds (e.g. using high-performance liquid chromatography [HPLC]). Furthermore, understanding how microbially derived carotenoids are obtained by hosts and the extent to which hosts may exploit them in their own physiological processes remain important targets for future research. More broadly, this work contributes to a growing body of evidence that microbial symbionts can play overlooked roles in shaping host traits with potential consequences for the ecology and evolution of their hosts. By uncovering the widespread occurrence and taxonomic diversity of carotenoid-producing bacteria within the gut microbiomes of wild sauropsids, our study establishes a foundation for investigating this hidden dimension of host-microbiome interactions.

## Supplementary Material

fiag100_Supplemental_Files
