# Supplementary material for: Hidden palette: culturing the sauropsid gut microbiome reveals a high prevalence and diversity of bacteria that undergo carotenoid biosynthesis
Source: FEMS Microbiol Ecol. 2026 Sep 7;102(10):fiag100. doi: 10.1093/femsec/fiag100 (PMC13599784; doi:10.1093/femsec/fiag100)
Supplement: Supplementary file 1 [file fiag100_supplemental_files.zip › FEMSME_FigS1_revision.pdf]

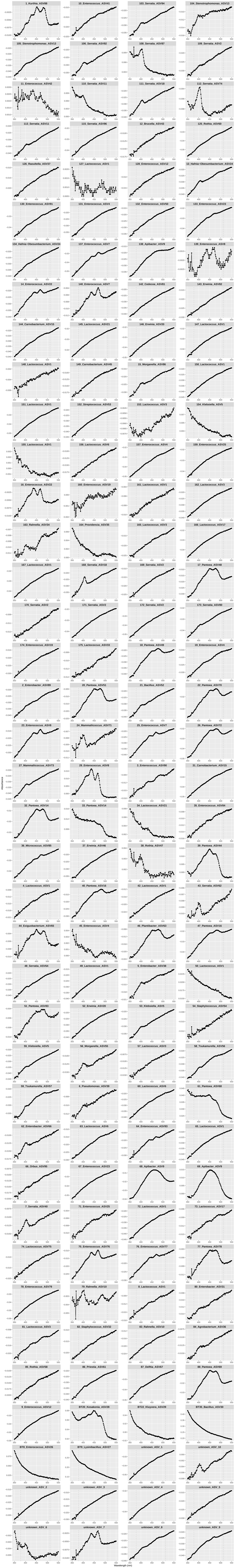
1_Kurthia_ASV88
10_Enterococcus_ASV81
103_Serratia_ASV94
104_Stenotrophomonas_ASV13
105_Stenotrophomonas_ASV13
106_Serratia_ASV92
108_Serratia_ASV87
109_Serratia_ASV2
11_Enterococcus_ASV42
110_Serratia_ASV11
111_Serratia_ASV18
112_Serratia_ASV74
113_Serratia_ASV11
115_Serratia_ASV96
12_Brucella_ASV43
125_Rothia_ASV60
126_Raoultella_ASV37
127_Lactococcus_ASV1
128_Enterococcus_ASV12
13_Hafnia-Obesumbacterium_ASV24
130_Enterococcus_ASV81
131_Enterococcus_ASV4
132_Enterococcus_ASV58
133_Enterococcus_ASV23
134_Hafnia-Obesumbacterium_ASV24
137_Enterococcus_ASV7
138_Apibacter_ASV9
139_Enterococcus_ASV8
14_Enterococcus_ASV22
140_Enterococcus_ASV7
142_Cedecea_ASV81
143_Erwinia_ASV82
144_Carnobacterium_ASV15
145_Lactococcus_ASV21
146_Erwinia_ASV20
147_Lactococcus_ASV1
148_Lactococcus_ASV1
149_Carnobacterium_ASV45
15_Morganella_ASV86
150_Lactococcus_ASV1
151_Lactococcus_ASV1
152_Streptococcus_ASV53
153_Lactococcus_ASV3
154_Klebsiella_ASV5
155_Lactococcus_ASV1
156_Lactococcus_ASV6
157_Enterococcus_ASV4
159_Enterococcus_ASV25
16_Enterococcus_ASV22
160_Enterococcus_ASV4
161_Lactococcus_ASV1
162_Lactococcus_ASV3
163_Rahnella_ASV34
164_Providencia_ASV35
165_Lactococcus_ASV3
166_Lactococcus_ASV17
167_Lactococcus_ASV1
168_Serratia_ASV18
169_Serratia_ASV2
17_Pantoea_ASV48
170_Serratia_ASV2
171_Serratia_ASV2
172_Serratia_ASV2
173_Serratia_ASV80
174_Enterococcus_ASV19
175_Lactococcus_ASV33
18_Pantoea_ASV49
19_Enterococcus_ASV4
2_Enterobacter_ASV89
20_Pantoea_ASV51
21_Bacillus_ASV52
22_Pantoea_ASV70
23_Enterococcus_ASV8
24_Mammaliicoccus_ASV71
25_Enterococcus_ASV7
26_Pantoea_ASV72
27_Mammaliicoccus_ASV73
29_Enterococcus_ASV8
3_Enterococcus_ASV90
31_Carnobacterium_ASV15
32_Pantoea_ASV14
33_Pantoea_ASV14
34_Lactococcus_ASV21
35_Enterococcus_ASV84
36_Micrococcus_ASV85
37_Erwinia_ASV46
38_Rothia_ASV47
39_Pantoea_ASV44
4_Lactococcus_ASV1
40_Pantoea_ASV16
42_Lactococcus_ASV1
43_Serratia_ASV62
44_Exiguobacterium_ASV65
45_Enterococcus_ASV4
46_Planifilum_ASV63
47_Pantoea_ASV64
48_Serratia_ASV64
49_Lactococcus_ASV1
5_Enterobacter_ASV39
50_Lactococcus_ASV1
51_Pantoea_ASV83
52_Erwinia_ASV20
53_Klebsiella_ASV5
54_Staphylococcus_ASV54
55_Klebsiella_ASV5
56_Morganella_ASV55
57_Lactococcus_ASV6
58_Tsukamurella_ASV56
59_Tsukamurella_ASV57
6_Pseudomonas_ASV28
60_Lactococcus_ASV6
61_Pantoea_ASV68
62_Enterobacter_ASV66
63_Lactococcus_ASV6
64_Enterococcus_ASV93
65_Lactococcus_ASV1
66_Orbus_ASV90
67_Enterococcus_ASV23
68_Apibacter_ASV9
69_Apibacter_ASV9
7_Serratia_ASV49
71_Enterococcus_ASV25
72_Lactococcus_ASV1
73_Lactococcus_ASV17
74_Lactococcus_ASV75
75_Enterococcus_ASV76
76_Enterococcus_ASV77
77_Pantoea_ASV78
78_Enterococcus_ASV79
79_Rahnella_ASV10
8_Lactococcus_ASV1
80_Enterobacter_ASV31
81_Lactococcus_ASV3
82_Staphylococcus_ASV32
83_Rahnella_ASV10
84_Agrobacterium_ASV36
85_Rothia_ASV58
86_Priestia_ASV61
87_Delftia_ASV67
88_Pantoea_ASV68
9_Enterococcus_ASV12
BT20_Kosakonia_ASV28
BT22_Kluyvera_ASV29
BT30_Bacillus_ASV30
BT8_Enterococcus_ASV26
BT9_Lysinibacillus_ASV27
unknown_ASV_1
unknown_ASV_10
unknown_ASV_2
unknown_ASV_3
unknown_ASV_4
unknown_ASV_5
unknown_ASV_6
unknown_ASV_7
unknown_ASV_8
unknown_ASV_9
Absorbance
Wavelength (nm)
