# Supplementary material for: Hidden palette: culturing the sauropsid gut microbiome reveals a high prevalence and diversity of bacteria that undergo carotenoid biosynthesis
Source: FEMS Microbiol Ecol. 2026 Sep 7;102(10):fiag100. doi: 10.1093/femsec/fiag100 (PMC13599784; doi:10.1093/femsec/fiag100)
Supplement: Supplementary file 1 [file fiag100_supplemental_files.zip › FEMSME_FigS2_revision.pdf]

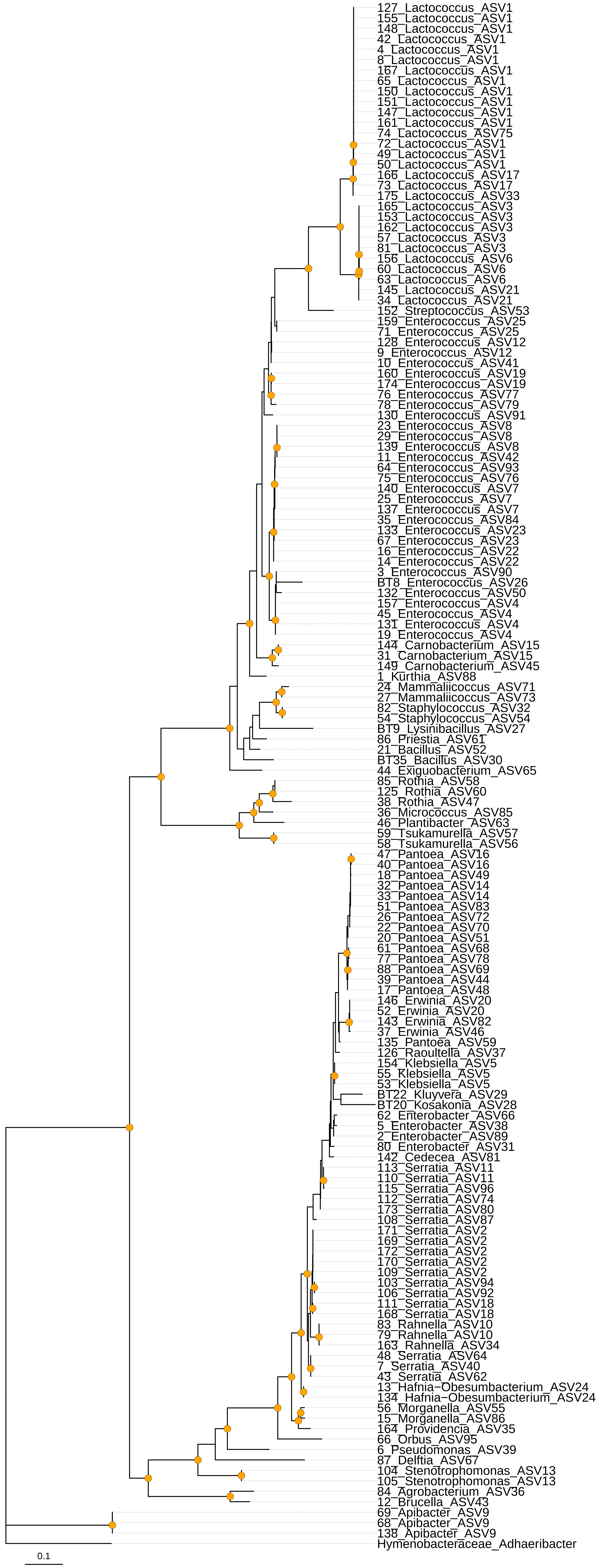
127_Lactococcus_ASV1
155_Lactococcus_ASV1
148_Lactococcus_ASV1
42_Lactococcus_ASV1
4_Lactococcus_ASV1
8_Lactococcus_ASV1
167_Lactococcus_ASV1
65_Lactococcus_ASV1
150_Lactococcus_ASV1
151_Lactococcus_ASV1
147_Lactococcus_ASV1
161_Lactococcus_ASV1
74_Lactococcus_ASV75
72_Lactococcus_ASV1
49_Lactococcus_ASV1
50_Lactococcus_ASV1
166_Lactococcus_ASV17
73_Lactococcus_ASV17
175_Lactococcus_ASV33
165_Lactococcus_ASV3
153_Lactococcus_ASV3
162_Lactococcus_ASV3
57_Lactococcus_ASV3
81_Lactococcus_ASV3
156_Lactococcus_ASV6
60_Lactococcus_ASV6
63_Lactococcus_ASV6
145_Lactococcus_ASV21
34_Lactococcus_ASV21
152_Streptococcus_ASV53
159_Enterococcus_ASV25
71_Enterococcus_ASV25
128_Enterococcus_ASV12
9_Enterococcus_ASV12
10_Enterococcus_ASV41
160_Enterococcus_ASV19
174_Enterococcus_ASV19
76_Enterococcus_ASV77
78_Enterococcus_ASV79
130_Enterococcus_ASV91
23_Enterococcus_ASV8
29_Enterococcus_ASV8
139_Enterococcus_ASV8
11_Enterococcus_ASV42
64_Enterococcus_ASV93
75_Enterococcus_ASV76
140_Enterococcus_ASV7
25_Enterococcus_ASV7
137_Enterococcus_ASV7
35_Enterococcus_ASV84
133_Enterococcus_ASV23
67_Enterococcus_ASV23
16_Enterococcus_ASV22
14_Enterococcus_ASV22
3_Enterococcus_ASV90
BT8_Enterococcus_ASV26
132_Enterococcus_ASV50
157_Enterococcus_ASV4
45_Enterococcus_ASV4
131_Enterococcus_ASV4
19_Enterococcus_ASV4
144_Carnobacterium_ASV15
31_Carnobacterium_ASV15
149_Carnobacterium_ASV45
1_Kurthia_ASV88
24_Mammaliicoccus_ASV71
27_Mammaliicoccus_ASV73
82_Staphylococcus_ASV32
54_Staphylococcus_ASV54
BT9_Lysinibacillus_ASV27
86_Priestia_ASV61
21_Bacillus_ASV52
BT35_Bacillus_ASV30
44_Exiguobacterium_ASV65
85_Rothia_ASV58
125_Rothia_ASV60
38_Rothia_ASV47
36_Micrococcus_ASV85
46_Plantibacter_ASV63
59_Tsukamurella_ASV57
58_Tsukamurella_ASV56
47_Pantoea_ASV16
40_Pantoea_ASV16
18_Pantoea_ASV49
32_Pantoea_ASV14
33_Pantoea_ASV14
51_Pantoea_ASV83
26_Pantoea_ASV72
22_Pantoea_ASV70
20_Pantoea_ASV51
61_Pantoea_ASV68
77_Pantoea_ASV78
88_Pantoea_ASV69
39_Pantoea_ASV44
17_Pantoea_ASV48
146_Erwinia_ASV20
52_Erwinia_ASV20
143_Erwinia_ASV82
37_Erwinia_ASV46
135_Pantoea_ASV59
126_Raoultella_ASV37
154_Klebsiella_ASV5
55_Klebsiella_ASV5
53_Klebsiella_ASV5
BT22_Kluyvera_ASV29
BT20_Kosakonia_ASV28
62_Enterobacter_ASV66
5_Enterobacter_ASV38
2_Enterobacter_ASV89
80_Enterobacter_ASV31
142_Cedecea_ASV81
113_Serratia_ASV11
110_Serratia_ASV11
115_Serratia_ASV96
112_Serratia_ASV74
173_Serratia_ASV80
108_Serratia_ASV87
171_Serratia_ASV2
169_Serratia_ASV2
172_Serratia_ASV2
170_Serratia_ASV2
109_Serratia_ASV2
103_Serratia_ASV94
106_Serratia_ASV92
111_Serratia_ASV18
168_Serratia_ASV18
83_Rahnella_ASV10
79_Rahnella_ASV10
163_Rahnella_ASV34
48_Serratia_ASV64
7_Serratia_ASV40
43_Serratia_ASV62
13_Hafnia−Obesumbacterium_ASV24
134_Hafnia−Obesumbacterium_ASV24
56_Morganella_ASV55
15_Morganella_ASV86
164_Providencia_ASV35
66_Orbus_ASV95
6_Pseudomonas_ASV39
87_Delftia_ASV67
104_Stenotrophomonas_ASV13
105_Stenotrophomonas_ASV13
84_Agrobacterium_ASV36
12_Brucella_ASV43
69_Apibacter_ASV9
68_Apibacter_ASV9
138_Apibacter_ASV9
Hymenobacteraceae_Adhaeribacter
0.1
