# Supplementary material for: Hidden palette: culturing the sauropsid gut microbiome reveals a high prevalence and diversity of bacteria that undergo carotenoid biosynthesis
Source: FEMS Microbiol Ecol. 2026 Sep 7;102(10):fiag100. doi: 10.1093/femsec/fiag100 (PMC13599784; doi:10.1093/femsec/fiag100)
Supplement: Supplementary file 1 [file fiag100_supplemental_files.zip › Table S1_20250119.docx]

Table S1. Summary of phenotype-based color categories. Each row includes the color name, a description of each color’s distinguishing characteristics, a representative image of an isolate for each color, and the individual identity that links the representative isolate to the labels on the phylogeny and absorbance assay curves.

| **Color** | **Description** | **Representative image** | **Identifier** |
| --- | --- | --- | --- |
| Pure white | Crisp, bright white |  | 74_Lactococcus_ASV75 |
| Snow white | Softer, fainter tone than pure white |  | 81_Lactococcus_ASV3 |
| Ivory white | Warm white, very slight hint of yellow |  | 111_Serratia_ASV18 |
| Off-white cream | Off-white with subtle pastel yellow, greater yellow undertone than ivory |  | 106_Serratia_ASV92 |
| Ivory cream | Warmer and richer yellow undertone than off-white cream |  | 62_Enterobacter_ASV66 |
| Beige cream | Contains more tan/light brown undertone than ivory cream |  | 53_Klebsiella_ASV5 |
| Pale yellow | Very light yellow, faded/pastel in color, subtle hints of white |  | 23_Enterococcus_ASV8 |
| Lemon yellow | Deeper and fuller shade of pale yellow |  | 77_Pantoea_ASV78 |
| Butter yellow | Crisp, light yellow |  | 61_Pantoea_ASV68 |
| Golden yellow | Strong, dark yellow |  | 69_Apibacter_ASV9 |
| Golden orange | Strong orange |  | 44_Exiguobacterium_ASV65 |
